# Contributions to Physiology

**Published:** 1850-01

**Authors:** 


### ARTICLE IV.

*Contributions to Physiology.*—By Bennett Dowler, M.D., Corresponding Member of the Academy of Natural Sciences of Philadelphia, etc. pp. 26. New-Orleans: 1849. (From the Author.)

This pamphlet is a separately covered copy of the article which originally appeared in our excellent exchange, the New Orleans Med. and Surg. Journal, detailing the author's experiments on the Alligator, as bearing upon the influence of the cerebro-spinal axis and nervous system generally, in the functions of volition, sensation, muscular motion, and the reflex Physiological doctrines of Dr. Marshall Hall.

As an animal for vivisection the one selected would appear better, because endowed with a higher organization, than frogs and turtles, which have been extensively used; but as the author readily admits, it is a very uncertain mode of investigating the physiology of man, to draw our conclusions from observations on reptiles. There is a gradation in physical organization, from the zoophyte, up to man; the links of which are almost imperceptible, yet the extremes are so far removed from each other, that no one proposes to deduce from the foot of the scale, evidence in reference to the complex functions of beings at or near the top of it. And as the gradation in the anatomy and physiology of the nervous system passes from the simple and scarcely perceptible ganglionic nervous centres situated in different parts of the worm, its only evidence of brain; up to the fully developed and exquisitely organized cerebro-spinal axis in man, we would expect sources of fallacy, almost innumerable, in applying observations upon an animal scarcely above the middle of the scale, to the explanation of the functions of the one at the very summit of it.

Notwithstanding these serious impediments in the way of arriving at the truth in reference to the functions of the human system, by observations made upon the lower animals, it is clear that we are not likely to ever have any better mode of investigation, and from what has been said, it will be at once inferred, that the higher in the scale the animal, the more nearly will the results correspond with what would take place in man under such experiments.

The distinguished author of this essay has on former occasions maintained the doctrine that the function of an organ, as contraction of muscles, &c., was dependent upon the peculiar adaptation of the organ, and not upon the nerves that ex end to it their peculiar influence.—That muscular contraction was not the result of reflex nervous action; in proof of which he adduced the fact that muscles will contract when struck upon, after being severed from the nervous centres, &c.

In the present article, he has gone farther, and shows by his experiments that sensation, will, and volition, are, in the alligator, independent of the brain, as the animal "decollated," (that is, with his head cut off,) for hours removed intelligently, irritating bodies, with his feet and legs, when applied to the headless trunk: and when placed so that the feet could not reach the irritant, fire, the body, deprived of forward motion, would roll *from* it. This would seem to show the body endowed with intelligence or mind, and but for the approach of the animal towards the foot of the scale of organization, where the brain is distributed through various parts of the system, would go far to disprove the doctrine of centralization, that the mind in man has a sanctum sanctorum, or especial abiding place, as the brain, and to prove that it pervades the whole frame like the spiritual body, as taught by Baron Swedenborg. But it is known as we ascend the scale, centralization increases, and step by step the cerebro-spinal axis acquires additional importance, as manifested by its increased size and influence over the various functions. The lowest order of animal life is so distributed through the system that the divided body forms two distinct living individuals; and as we ascend higher, we find tenacity o life diminish, and the severed parts dying moie and more speedily.

We quote one of the several experiments reported in the paper, authenticated by the presence of Dr. Cartwright, Prof. Forshey, and five other gentlemen, four of whom were physicians, made upon an alligator about three feet long:

* * * "The decollation was not followed by a projecting stream of blood, as is usual; no ligature was applied to the great artery of the neck. The dull hatchet used in severing the spine of the neck, had probaby bruised the artery as in torison and gun-shot wounds. Hence the hemorrhage was not great, though considerable.

I carried the handle of the knife towards the eye, to ascertain whether it would wink; whereupon the ferocious, separated head, sprang up from the table with great force, at me, passing very near my breast, which received several drops of blood; it alighted upon the floor, from six to eight feet distant from its original position! It missed me, because I was standing at the side, and not in front of the head. Although, I have examined carefully, all the muscles of the head, I cannot find one that accounts for this feat of combative muscular motion. The angles of the mouth recede so much in this animal, that after decollation, including the medulla oblongata, the head seemed almost like two separate pieces,—the superior and the inferior maxillary bones, being joined chiefly by the great masseter muscles, for only a short distance. These great muscles, (the masseters,) which are curved, having their concavity anteriorly, are adapted only to vertical action, as in biting—the great muscles of the tongue act backward and upward against the palatine region:—whence then this quick, violent, forward motion, or rather, as in this case, diagonal leap of six or eight feet—for the head deviated to the left, where I was standing, evidently with the intention of biting me? Tne trunk, in this, as in all cases, possessed no power of forward motion. This curious fact with respect to decapitated animals, noticed by M. Magendie, and other vivisectors, has been attributed to the *loss of the cerebellum;* but whether this loss of forward motion in the alligator, be owing to a division of the spine, and great muscles, or to the separation of the larger or smaller brain, or both, is not very evident, yet the fact which I have noticed respecting the forward motion of the separated head, is, perhaps, a circumstance favorable to this view. That a voluntary, spontaneous and powerful motion,—in fact a diagonal leap, should be performed by the separated head, must therefore appear astounding to one acquainted with the muscular organization. It is difficult to understand, how the cerebellum could thus act alone.

"For about two hours, the headless trunk of the last mentioned alligator, exhibited such phenomena as are usually attributed to the brain, namely, sensation, volition, and intelligential motion, as tested by the application of bits of ignited paper, wounds, and the like, whereupon, the usual indicants of pain were elicited with promptness and precision: it trembled, receded, rolled over, curved, placed its limbs accurately to the exact spot, and removed the offending cause. In certain places this was exceedingly difficult, as on the spine between or near the shoulders, or hips. It always used the limb best adapted for the purpose. If the fire was too remote, as when applied to the tail, the whole body was thrown into the most favorable position, for the purpose of reaching and removing the same. If the fire was placed on the table, in a position to annoy, yet without touching, the animal, as if endowed with sight, reached, and always accurately, to the exact spot, and either extinguished the fire, or removed it. As upon former occasions, if the animal found that the fire was continued at the same spot, and that it could not remove it, which was sometimes the case, owing to continuous, or repeated applications, and carefully manœuvring, it curved the body—scratched violently, manœuvred skillfully, and then as a last resort, rolled quite over, laterally, always *from*, never *towards* the fire and operator.

"After these experiments had progressed for some time, Dr. Cartwright desired me to cut off the neck close to the shoulders. This was done, but the intelligential, sensational, and volitional motions continued as before."

We are equally at a loss with the author, to account for that leap of the head.

As the principal support of the reflex doctrine is drawn from experiments made upon animals equally low with the alligator, one of the experiments of our author militates strongly against it. In this experiment, the spinal cord was completely severed in the neck and midway between the shoulders and hips, after which the animal acted with perfect harmony between all its parts—"It saw, heard, felt, defended itself, showed anger, fear, and even friendly attention to its keeper, a black boy." The fore legs and hind legs acted mutually in removing a pricking body above the division in the cord.

The arguments of the author are forcible, and his familiarity with the subject apparent from his ready use of the opinions of others, which he handles without gloves. E.

